# Characterizing dopaminergic neuron vulnerability using genome-wide analysis

**DOI:** 10.1093/genetics/iyab081

**Published:** 2021-05-26

**Authors:** Jacinta Davis, Claire Da Silva Santos, Narda Caudillo Zavala, Nicholas Gans, Daniel Patracuolla, Monica Fehrenbach, Daniel T Babcock

**Affiliations:** Department of Biological Sciences, Lehigh University, Bethlehem, PA 18015, USA

**Keywords:** *Drosophila*, neurodegneration, dopamine

## Abstract

Parkinson’s disease (PD) is primarily characterized by the loss of dopaminergic (DA) neurons in the brain. However, little is known about why DA neurons are selectively vulnerable to PD. To identify genes that are associated with DA neuron loss, we screened through 201 wild-caught populations of *Drosophila melanogaster* as part of the Drosophila Genetic Reference Panel. Here, we identify the top-associated genes containing single-nucleotide polymorphisms that render DA neurons vulnerable. These genes were further analyzed by using mutant analysis and tissue-specific knockdown for functional validation. We found that this loss of DA neurons caused progressive locomotor dysfunction in mutants and gene knockdown analysis. The identification of genes associated with the progressive loss of DA neurons should help to uncover factors that render these neurons vulnerable in PD, and possibly develop strategies to make these neurons more resilient.

## Teaser

Using *Drosophila*, Davis *et al*. identify novel factors that render dopaminergic neurons vulnerable to degeneration. As dopaminergic neurons are selectively vulnerable in Parkinson’s Disease, these results provide insight into the development of potential therapeutic targets to protect these vulnerable neuronal populations.

## Introduction

Parkinson’s disease (PD) is the second most prevalent neurodegenerative disease and the most prominent movement disorder globally (Beitz 2014). The most common symptoms of PD cover a variety of locomotor dysfunctions, including tremors, bradykinesia, and propulsive gait. These locomotor problems associated with PD are caused by the loss of dopaminergic (DA) neurons within the substantia nigra (Bayersdorfer *et al.* 2010). It remains unclear, however, why these particular neurons are selectively vulnerable to PD. With an aging population and a lack of cures or viable treatment options, the prevalence rates of PD are expected to increase drastically over the next several decades (Xie *et al.* 2018). As such, understanding the factors that render these types of neurons vulnerable is critical for identifying potential therapeutic targets in this disease.

*Drosophila* is a well-established model system to study neurodegenerative diseases such as PD (Feany and Bender 2000; Riemensperger *et al.* 2013; Cunningham *et al.* 2018; Reeve *et al.* 2018; Xie *et al.* 2018). The *Drosophila* brain contains approximately 100 DA neurons that are organized into distinct clusters and are amenable to quantitative analysis (Mao and Davis 2009). Additionally, *Drosophila* DA neurons are vulnerable to many of the same factors seen in patients with PD, including expression of human *α-Synuclein* (Periquet *et al.* 2007), mutations in genes such as *PINK1* and *parkin* (Greene *et al.* 2003; Yang *et al.* 2006), and exposure to chemicals such as rotenone and paraquat (Coulom and Birman 2004). Finally, this loss of DA neurons is associated with locomotor dysfunction (Riemensperger *et al.* 2013), making *Drosophila* a useful model for understanding the vulnerability of these neurons.

In addition to transgenic and mutagenesis screens that are commonly associated with *Drosophila*, recent work has highlighted the examination of genetic variation within natural populations as a powerful tool for characterizing genetic traits (MacKay *et al.* 2012). One such toolkit is the Drosophila Genetics Reference Panel (DGRP), a collection of isogenic stocks from 201 wild-caught lines of *Drosophila melanogaster* (MacKay *et al.* 2012). This method has been used to identify single-nucleotide polymorphisms (SNPs) associated with several quantitative traits that vary in nature (Chow *et al.* 2016; Lavoy *et al.* 2018; Katzenberger *et al*. 2015), providing a useful option for performing forward genetic analysis.

Here, we demonstrate that there is natural variance in the maintenance of DA neurons in aged flies across genetic backgrounds. We performed an unbiased genome-wide screen and identified several genes containing SNPs strongly associated with the maintenance of DA neurons. Functional validation of these candidate genes through RNAi-mediated knockdown, along with mutant analysis, reveals that loss of function of these genes results in a progressive, age-dependent loss of DA neurons primarily acting in a cell autonomous fashion. Additionally, climbing analysis reveals a progressive loss of locomotor function in these mutants. As nearly all of these candidate genes have clear human homologs, our results should provide additional insight into the mechanisms responsible for maintaining DA neurons with age and disease.

## Methods

### Fly stocks and husbandry

Fly stocks were maintained at 25°C on standard *Drosophila* media. Flies used for experimental analysis were collected upon eclosion, separated by sex, and aged for 21 days at 29°C. For aging experiments, flies were transferred to fresh food every 2 days. The following stocks were obtained from the Bloomington Drosophila Stock Center: DGRP stocks (MacKay *et al.* 2012), *Sesn^BG01215^* (Bellen *et al.* 2004), *Sesn^MI03130^* (Bellen *et al.* 2004), *tweek^MI09550^* (Bellen *et al.* 2004), *tweek^1^* (Verstreken *et al.* 2009), *tweek^2^* (Verstreken *et al.* 2009), *Lim3^1^* (Thor *et al.* 1999) *Lim3^2^* (Black *et al.* 1987), *Trf2^G0039^* (Bashirullah *et al.* 2007), *kirre^rP298-G4^* (Kim *et al.* 2015), *mgl^G17930^* (Bellen *et al.* 2004), *CG42339^M110691^* (Nagarkar-Jaiswal *et al.* 2015), *plexus* (Matakatsu *et al.* 1999), *w^1118^ Oregon-R*, *TH-Gal4* (Friggi-Grelin *et al.* 2003), *w^1^* (Bingham *et al.* 1981), *yw67c23* (Biessmann 1985), *UAS-Dicer2* (Dietzl *et al.* 2007), *UAS-Tow^IR^* (Ni *et al.* 2011), *UAS-CG42339^IR^* (Ni *et al.* 2011), *UAS-Megalin^IR^* (Ni *et al.* 2011), *UAS-Trf2^IR^* (Ni *et al.* 2011), *UAS-Kirre^IR^* (Ni *et al.* 2011), *UAS-Tweek^IR^* (Ni *et al.* 2011), *UAS-Sestrin^IR^* (Ni *et al.* 2011), *UAS-Lim3^IR^* (Ni *et al.* 2011), *P{UAS-mCherry.VALIUM10}attP2*, *UAS-Luciferase* (Perkins *et al.* 2015), *P{CaryP}attP40* (Markstein *et al.* 2008), and *nsyb-Gal4* (Pauli *et al.* 2008). The following stocks were obtained from the Vienna Drosophila Resource Center (VDRC): *UAS-PlexusIR* (ID: 40524) (Dietzl *et al.* 2007) *Control vector for KK collection (ID: 60100)* (Vissers *et al.* 2016).

### Immunohistochemistry

Brains were dissected and stained as previously described (Babcock and Ganetzky 2015). Briefly, brains were dissected in 1× PBS and fixed immediately in 4% paraformaldehyde for 20 minutes at room temperature. Samples were then washed using PBS with 0.3% Triton x-100 (PBST) for 5 minutes each at room temperature a total of five times. Brains were then placed in blocking buffer (PBS, 0.2% Triton X-100, and 0.1% normal goat serum) for a minimum of 1 hour at 4°C. Primary antibody was then added to each sample for 48 hours at 4°C. Antibody was removed from samples and brains were then washed with PBST four times for 5 minutes each at room temperature. Secondary antibody was added to the samples and left to incubate for 2 hours at room temperature in the dark. Samples were washed with PBST 4 times and then mounted with Vectashield. Slides were imaged immediately or preserved at −20°C. The primary antibody used was rabbit anti-tyrosine hydroxylase (1:100, AB152, Millipore). The secondary antibody used was Alexa Fluor 488 goat anti rabbit (1:200, Fisher Scientific).

### Dopaminergic neuron quantification

DA neurons were counted using a Nikon Eclipse Ni-U fluorescent microscope equipped with a 20× objective. DA neurons located in the PPL1, PPM1/2, and PPM3 were counted for both hemispheres for each brain sample. A minimum of 10 brains were analyzed for each genotype and sex in every experiment. Male and female samples were analyzed separately, and only combined if there were no statistically significant differences between sexes. For mutants of genes located on the X chromosome, hemizygous males were analyzed. Since no differences were observed between sexes in any of our measurements, the data presented represents combined results. Experiments were performed in triplicates and were scored blindly with regard to genotype or condition. Average values for each measurement are listed in Supplementary Table S3.

### Image analysis

Images were taken using a Zeiss LSM 880 confocal microscope. 3.5 μm optical slices were taken using a 20× objective for all images taken. Confocal stacks were flattened using ImageJ software. Different clusters of DA neurons were false-colored for organization using ImageJ software. Scale bars for all images are at 25 μm and listed in the figure legends. Brightness and contrast were adjusted equally for all images used in each experiment using Adobe photoshop.

### Locomotor behavior

Adult flies were collected shortly after eclosing and separated by sex. Groups for each genotype consisted of 10 male or female flies, with a total of 80–100 flies for each genotype. Similar to the DA neuron quantification, we used hemizygous males for X chromosome mutations. All other groups had males and females combined since we saw no differences in sex. Flies were aged for 3 days or 21 days at 29°C and were transferred to fresh food every 2 days. The climbing assay starts with transferring each group into a tube consisting of two glass vials connected at the open ends (total diameter, 2.5 cm; total height, 20 cm). Each group was allowed to acclimate to the glass vials for a total of 5 minutes. The climbing index for each group was defined as the percentage of flies that climbed to a 8 cm mark in the glass vials within 20 seconds of tapping the glass vial onto a mouse pad. Three trials were carried out for each group of flies. Between each trial the flies were allowed to recover for at least 1 minute. Average values for each measurement are listed in Supplementary Table S3.

### Statistical analysis

For both the climbing index and DA neuron loss measurements, a two-way ANOVA was used with multiple comparisons and Tukey’s alignments using Graphpad Prism (Graphpad Software, Inc.). SNPs associated with the loss of DA neurons were identified using the Drosophila Genetics Reference Panel Freeze 2 GWAS webtool (http://dgrp2.gnets.ncsu.edu/) (Huang *et al.* 2014).

### RNA isolation and qPCR

RNA was isolated and qPCR was carried out as previously described (Sidisky *et al.* 2021). We adapted this protocol by using 40 heads per each condition as samples for RNA isolation. RNA extraction was performed using Trizol (Invitrogen) and phenol chloroform for each sample according to the manufacturer’s instructions. The RNA samples were then treated using the NEB RNA Clean-up kit (NEB T2030) according to the manufacturer’s instructions. The qRT-PCR experiments were completed using an ABI7300 Real-Time thermocycler and Sybergreen powerup master mix (Applied Biosystems). Each sample was repeated in triplicate form allowing us to gather average (C_t_) values. We used Actin 5c as our internal control (Dalui and Bhattacharyya 2014). In order to calculate the fold change in expression of each gene within the mutants and RNAi knockdown relative to WT, we used the 2^(−ΔΔCT)^ method. Primer sequences are listed in Supplementary Table S4 along with the references/sources for each.

### Data availability

The authors affirm that the conclusions of the article are present within the article, figures, and tables. Supplemental Material available at figshare: https://doi.org/10.25386/genetics.14519403.

## Results

### An unbiased genome-wide screen reveals novel genes associated with the maintenance of dopaminergic neurons

**Figure 1:**
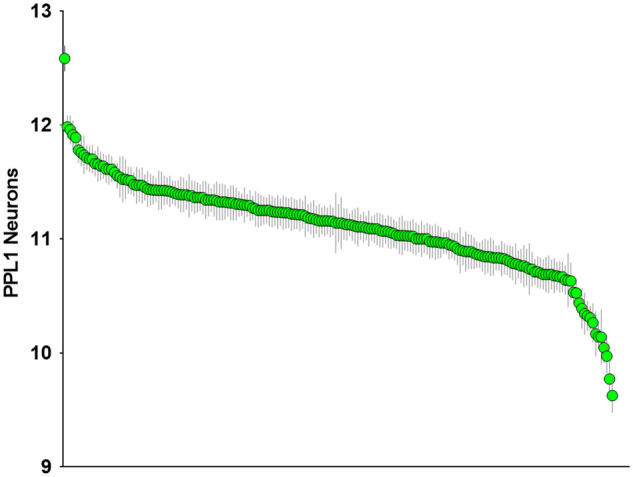
Vulnerability of DA neurons varies across genetic backgrounds. Green dots measure the average number of PPL1 neurons per cluster in each of the 201 DGRP fly stocks at Day 21. Genes harboring SNPs most significantly associated with fewer DA neurons are listed in Table 1.

We investigated the natural variance of the number of DA neurons in wild-caught *Drosophila melanogaster* populations to identify genes responsible for maintaining these neurons. We specifically examined the DA neurons in the protocerebral posterior lateral 1 (PPL1) cluster (Mao and Davis 2009) within aged brains. We focused on the PPL1 cluster due to a significant loss of neurons in this cluster previously found in *parkin* mutants, while other clusters remained unaffected (Whitworth *et al.* 2005). We found significant variability in the amount of PPL1 neurons among 201 wild-caught lines that were aged to 21 days at 29°C, with averages ranging from 12 to 9 (Figure 1, Supplementary Tables S1 and S2). These results suggest that the number of DA neurons in aged flies is a phenotype that varies across genetic backgrounds. These data were submitted to the Drosophila Genetics Reference Panel 2 to identify SNPs associated with this variance (Huang *et al.* 2014). We identified nine genes harboring SNPs that were highly associated with the variation of DA neurons in the PPL1 cluster (Table 1, Supplementary Table S2). These genes represent diverse cellular functions, further highlighting the complexity of neuronal maintenance. Importantly, eight of these nine genes have clear human homologs, demonstrating that understanding the role of these genes in regulating DA neuron viability will likely be relevant to human health and disease.

**Table 1:** Top candidate genes

Rank	Candidate gene	SNP	*P*-value	Human ortholog	OMIM
1	*Tow*	3L: 6937595	2.56E−07	C1orf21	—
2	*CG42339*	X: 10933694	4.32E−07	SBSPON	—
3	*megalin*	X: 9318757	8.05E−07	LRP2	600073
4	*plexus*	2R: 18423826	1.06E−06	None	—
5	*Trf2*	X: 8325561	1.78E−06	TBPL1	605521
6	*kirre*	X: 2800634	2.77E−06	KIRREL	607428
7	*tweek*	2L: 16900031	3.24E−06	KIAA1109	611565
8	*sestrin*	2R: 19614642	3.40E−06	SESN3	607768
9	*Lim3*	2L: 19086842	9.20E−06	LHX3	600577

### Functional validation of candidate genes using tissue-specific RNAi knockdown

**Figure 2:**
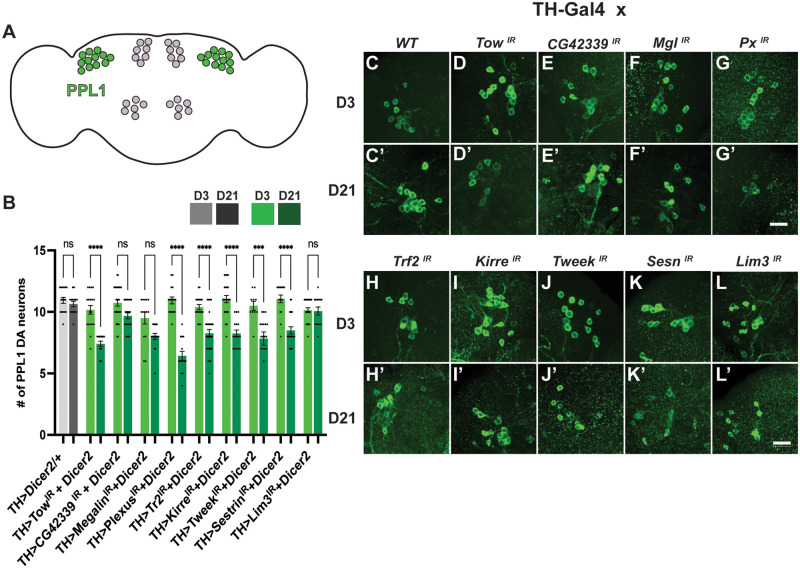
Loss of DA neurons in the PPL1 cluster is significant upon knockdown of candidate genes. (A) Posterior lateral protocerebrum (PPL1) neurons labeled in green (B) Quantitative analysis of DA neuron loss observed in the tissue-specific knockdown of *Tow*, *Plexus*, *Trf2*, *Kirre*, *Tweek*, *Sestrin* at Day 21. (C–L) D3 qualitative images of PPL1 neurons stained with Tyrosine Hydroxylase (TH). (C’–L’) D21 qualitative images of PPL1 neurons stained with TH in comparison to D3. ***P < 0.001, ****P < 0.0001. NS, not significant. Scale bar = 25 µm for all confocal images.

To functionally validate the genes identified in our screen, we knocked these genes down in DA neurons using RNAi. We found that when six of these genes are knocked down in DA neurons using a Tyrosine hydroxylase driver *TH-Gal4* (Friggi-Grelin *et al.* 2003) and *UAS-Dicer2* (Coulom and Birman 2004), there are fewer neurons compared to wild-type controls (Figure 2), demonstrating similar results from our DGRP analysis. To determine whether the lower number of neurons is due to a developmental defect as opposed to a progressive loss of neurons, we also quantified the number of PPL1 neurons at day 3. Each condition with fewer PPL1 neurons at day 21 had significantly greater numbers of neurons at day 3, suggesting that knocking down these genes in DA neurons results in a progressive, age-dependent loss of neurons (Figure 2B). The tissue-specific knockdown of these genes also reveals that they act in a cell-autonomous fashion to maintain DA neurons. There were three genes that did not show significant DA neuron loss at day 21 when knocked down in DA neurons: *CG42339*, *Megalin*, and *Lim3* (Figure 2, E’, F’, L’)*.* It is possible that the level of knockdown was insufficient to produce a measurable phenotype. To evaluate the strength of the knockdown, we performed qPCR to analyze the transcript levels for each gene upon expression of the RNAi transgenes. While transcript levels of both *CG42339* and *Megalin* are noticeably reduced using RNAi, we did not observe a clear knockdown of *Lim3* (Supplementary Figure S1). We also determined that the UAS-RNAi transgenes themselves did not have an impact on neuronal loss (Supplementary Figure S2). Thus, the lack of neuronal loss using RNAi for *Lim3* could be due to the inefficiency of the transgene. For *CG42339* and *Megalin*, it is possible that the drastic reduction in expression using RNAi is still insufficient to cause neuronal loss. Another possibility is that neither of these genes are required directly within DA neurons to maintain these cells.

To further test whether the reduced number of TH+ neurons is indeed a loss of neurons, we knocked down our candidate genes using RNAi while co-expressing the fluorescent marker mCherry. We observed a significant decrease in the number of mCherry+ PPL1 neurons under the same conditions using TH staining (Supplementary Figure S3). These results suggest that knocking down candidate genes in DA neurons results in the loss of neurons rather than a specific reduction in TH staining. The one exception for this experiment was *Trf2*. As combinations of fluorescent markers with this RNAi transgene were very unhealthy, we were unable to quantify PPL1 neurons in this manner. Thus, we cannot rule out the possibility that *Trf2* knockdown leads to a specific decrease in TH staining without neuronal loss.

Although our screen was focused on the PPL1 cluster of DA neurons, we also examined whether other clusters of DA neurons were similarly vulnerable under the same conditions. To determine whether the variability of PPL1 neurons within the DGRP stocks would show a similar pattern in other DA neuron clusters, we reexamined brain samples from DGRP lines showing the highest as well the lowest average number of PPL1 neurons and measured the number of neurons within the protocerebral posterior medial 1 and 2 (PPM1/2) and protocerebral posterior medial 3 (PPM3) clusters at day 21. Interestingly, we found no significant difference in the amount of DA neurons between any of these DGRP lines for the PPM1/2 and PPM3 clusters (Supplementary Figure S4). Thus, the variation in the number of PPL1 neurons from our screen does not appear to correlate with natural variation in other clusters. It is currently unclear whether examination of all DGRP stocks would reveal natural variation in the maintenance of DA neurons within these clusters.

**Figure 3:**
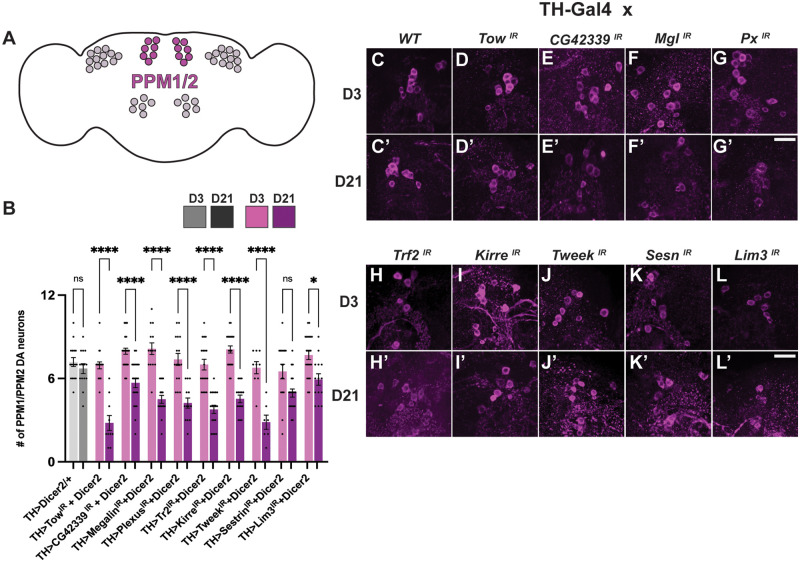
Loss of PPM1/2 DA neurons is significant upon knockdown of candidate genes. (A) Posterior medial protocerebrum (PPM1/2) labeled in magenta. (B) Quantitative loss of DA neurons was found upon *CG42339*, *Megalin*, *Trf2*, *Kirre*, and *Lim3* knockdown. (C–L) D3 qualitative images of DA neurons located in the PPM1/2 clusters. (C’-L’) D21 qualitative images of DA neurons in comparison to D3 are stained with TH. *P < 0.05, ****P < 0.0001. NS, not significant. Scale bar = 25 µm for all confocal images.

While the natural variation in DA neuron maintenance of PPL1 neurons did not directly correlate with maintenance of other clusters among the DGRP lines, we also examined whether RNAi-mediated knockdown of our nine candidate genes in all DA neurons impacted the viability of these other clusters of DA neurons. We observed a significant progressive loss of DA neurons located in the PPM1/2 clusters upon knockdown of each of the candidate genes except *Sestrin* (Figure 3B). Representative images show that there is a progressive loss of DA neurons for these eight genes, not including *Sestrin* (Figure 3, C’–L’). These results reveal a remarkable similarity between genes required to maintain both PPL1 and PPM1/2 neurons. It is unclear why no variation was observed in this cluster between the top and bottom hits from the DGRP screen, but RNAi-mediated knockdown of the candidate genes certainly has similar effects in these clusters. The two differences found between PPL1 and PPM1/2 clusters involve *Sestrin* and *Lim3*. Despite the overwhelming similarities between these clusters, perhaps certain DA neurons rely more heavily on particular gene expression to maintain viability with age.

Similar to our investigation of PPL1 neurons, we investigated the RNAi lines alone and the empty vectors for each construct driven with *TH-Gal4* for any possible neuron loss in the PPM1/2 cluster. We found no significant loss across all conditions at day 3 and day 21 (Supplementary Figure S5). To further verify that the loss of PPM1/2 neurons is not due to a loss of TH staining, we used expression of *UAS-mCherry* with TH to drive each RNAi line and *UAS-Dicer2*. As with the PPL1 cluster, the loss of mCherry signal was consistent with the loss of TH staining in nearly every condition (Supplementary Figure S6). The two exceptions were again *Sestrin* and *Lim3*. This could be explained by differences in the exact number of DA neurons labeled using a TH antibody in comparison to transgenic expression using *TH-Gal4*, as previously reported (Mao and Davis 2009). Together, the differences and similarities between the PPL1 and PPM1/2 results may indicate that different clusters are more or less vulnerable to changes in viability with age.

**Figure 4:**
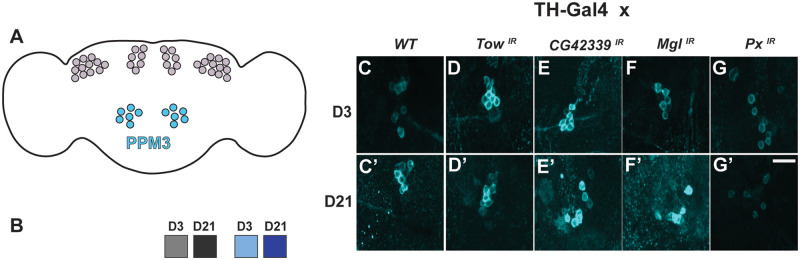
No significant loss of DA neurons in the PPM3 cluster upon knockdown of candidate genes. (A) Posterior medial protocerebrum (PPM3) labeled in blue. (B) No significant loss of DA neurons in the PPM3 cluster was observed when all nine genes were individually knocked down. (C–L) Qualitative images of the PPM3 cluster at D3 for each gene knockdown. (C’–L’) Qualitative images of the PPM3 cluster at D21 compared to D3. NS, not significant. Scale bar = 25 µm for all confocal images.

Finally, we investigated DA neuronal maintenance in the PPM3 cluster in a similar manner to that of the other clusters. Interestingly, knockdown of the candidate genes did not result in any significant loss of DA neurons in the PPM3 cluster from day 3 and day 21 (Figure 4B). Representative images depicted that there is no significant loss of DA neurons when quantified at each time point (Figure 4, C’–L’). When testing the RNAi lines alone and empty vectors driven with *TH-Gal4* we saw no significant loss of DA neurons (Supplementary Figure S7). Images show no loss of neurons on day 21 when compared to day 3 for all groups (Supplementary Figure S7). We also found that when using mcherry with *TH-Gal4* for all knockdown lines coupled with UAS-dicer2 still showed no loss of DA neurons in the PPM3 cluster at day 21 (Supplementary Figure S8). These results suggest that PPM3 DA neurons are more resilient to the loss of these candidate genes when compared to the PPL1 and PPM1/2 clusters.

### Mutant alleles of the nine genes shows varied loss of DA neuronal clusters

**Figure 5:**
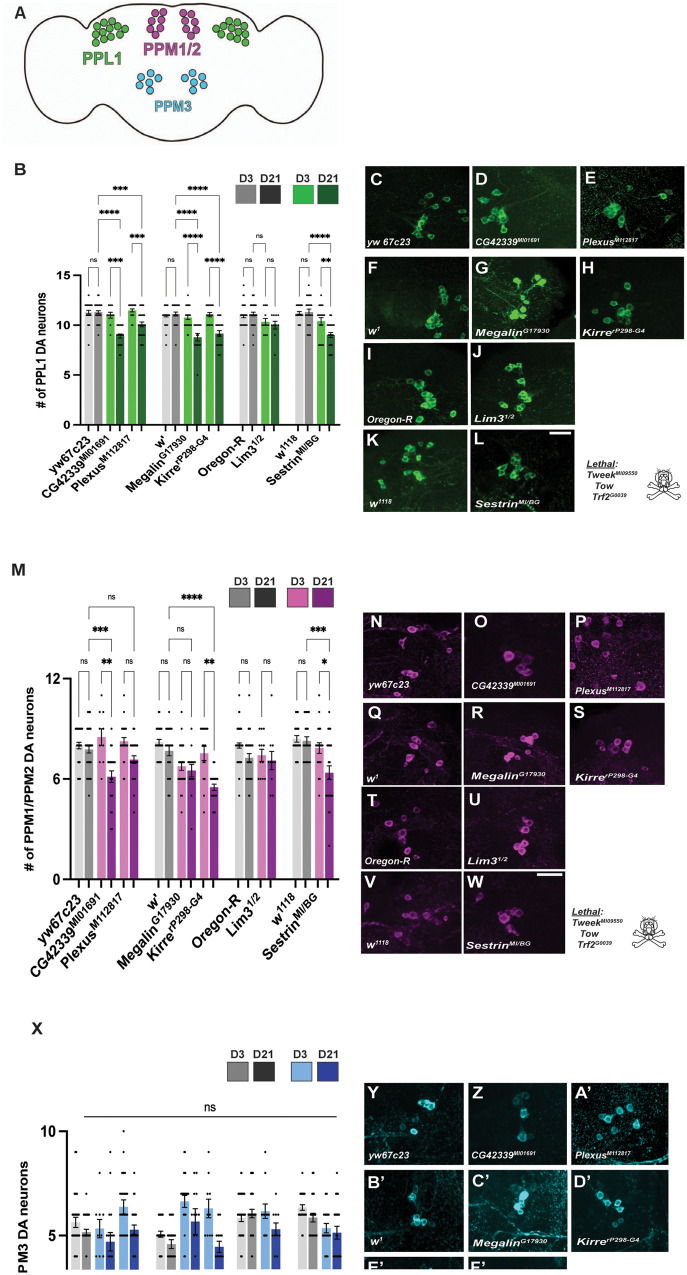
Significant loss of DA neurons for select genes in clusters PPM1/2 and PPL1. (A) Average number of neurons per cluster: PPL1, PPM1/2 and PPM3 (B) Quantitative analysis of each mutant line at an early and late time point compared to each mutants specific control background. (C–L) PPL1 neurons are progressively lost in *CG42339*, *Plexus*, *Megalin*, *Kirre*, and *Sestrin*. *Lim3* mutants do not show a significant loss. (M) Quantitative analysis of DA neurons in the PPM1/2 shows that mutations in *CG42339*, *Kirre* and *Sestrin* causes a significant loss at day 21 while the other three genes do not. (N–W) Day 21 images of the PPM1/2 cluster for the six non-lethal genes and controls. (X) No significant loss of DA neurons at Day 21 found in the PPM3 cluster. (Y–H’) Qualitative images of the PPM3 cluster for the control and experimental groups. *Tweek, *Tow*, and *Trf2* mutants were lethal and not homozygous viable. *P < 0.05, **P < 0.01, ***P < 0.001, ****P < 0.0001. NS, not significant. Scale bar = 25 µm for all confocal images.

In addition to the tissue-specific knockdown of our candidate genes using RNAi, we also examined DA neuron maintenance using publicly available mutant stocks of these genes. Mutants of the genes *Trf2*, *Tweek*, and *Tow* were not homozygous viable in our experiments. For the remaining mutant stocks, we quantified DA neurons in each mutant compared to the genetic background. (Figure 5). Among the six remaining genes *CG4233*, *Plexus*, *Megalin*, *Kirre*, and *Sestrin* mutants showed a significant loss of DA neurons in the PPL1 cluster at day 21 while *Lim3* mutants did not (Figure 5, B–L). *CG42339*, *Kirre*, and *Sestrin* all depicted a significant progressive loss of DA neurons in the PPM1/2 cluster (Figure 5, M, O, S, W). There was no observed loss of PPM1/2 neurons for *Megalin* and *Lim3* (Figure 5, M, R, U). Similar to our results using RNAi knockdown, there was no significant loss of DA neurons within the PPM3 cluster at day 21 (Figure 5, X–H’). The neuronal loss seen in our mutant analysis was not as significant compared to the RNAi-mediated knockdown. One possible explanation for this result is that the mutations we tested were not severe enough to cause DA neuron loss. Therefore, we tested the levels of transcripts for each mutant line compared to wildtype by qPCR. We found that there was a downregulation in the relative gene expression of each mutant line except *Lim3* and a weak reduction in *Kirre* (Supplementary Figure S9). Thus, these genes may play a role in varying biological mechanisms and may cause dysfunction upon other processes. A more comprehensive analysis of mutations in each of these genes may provide more detailed information regarding the role of these genes in DA neuron maintenance.

### Mutants and RNAi knockdown of candidate genes show locomotor defects

**Figure 6:**
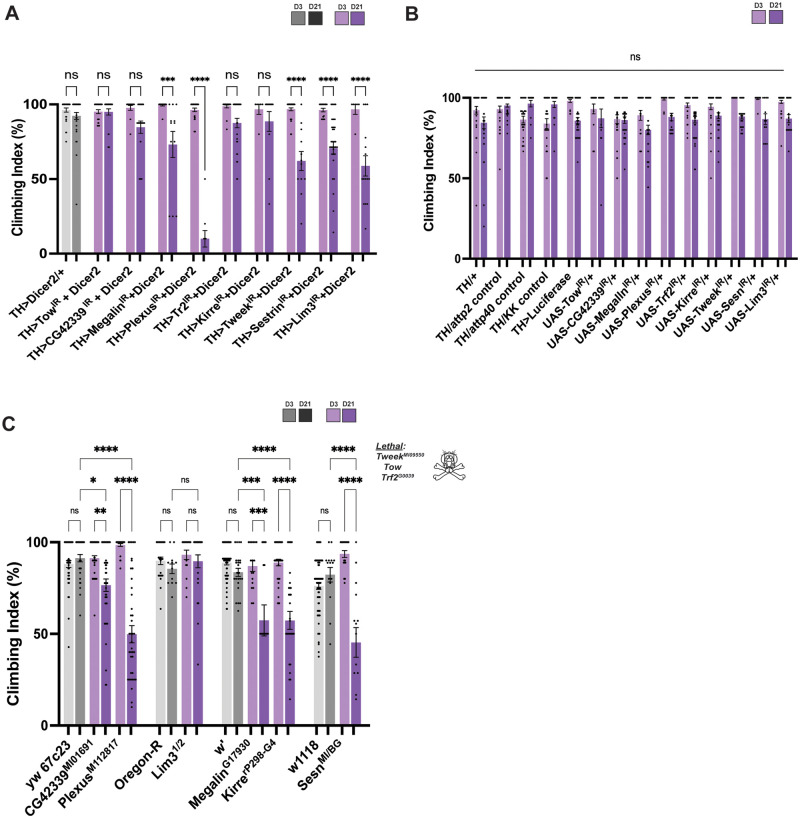
Progressive locomotor dysfunction found when candidate genes are not maintained. (A) Tissue-specific knockdown of *Megalin*, *Plexus*, *Tweek*, *Sestrin*, and *Lim3* in DA neurons causes climbing defects at Day 21. (B) No significant dysfunction was found between all genotypes for the control vectors and the knockdown lines alone. (C) Mutations in *CG42339*, *Plexus*, *Megalin*, *Kirre*, and *Sestrin* cause significant locomotor dysfunction at day 21. While there is no detected locomotor dysfunction for *Lim3* mutants. *P < 0.05, **P < 0.01, ***P < 0.001, ****P < 0.0001. NS, not significant.

Previous studies investigated locomotor defects in *Drosophila melanogaster* as a behavioral assay for neurodegeneration (Riemensperger *et al.* 2013). We used this assay to further validate the RNAi knockdown and mutagenesis of these nine genes. We found that when five of the nine genes are knocked down in the DA neurons, there is a significant progressive locomotor defect. The genes that showed this defect at day 21 are: *Megalin*, *Plexus*, *Tweek*, *Sestrin*, and *Lim3* (Figure 6A). We found that the RNAi lines alone and the empty vectors driven by *TH-Gal4* showed no locomotor dysfunction (Figure 6B). We also investigated climbing defects with the mutant stocks. We discovered that mutations in *CG42339*, *Plexus*, *Megalin*, *Kirre*, and *Sestrin* displayed significant locomotor dysfunction at day 21 (Figure 6C). The only mutants that did not have a reduced climbing index at day 21 was *Lim3.* Interestingly, we found that when *Kirre* and *CG42339* were knocked down in DA neurons there is no locomotor dysfunction observed despite the consistent loss of DA neurons. This discrepancy provides further insight into the vulnerability of specific DA neuron populations and their relationship to locomotor behavior.

## Discussion

**Figure 7:**
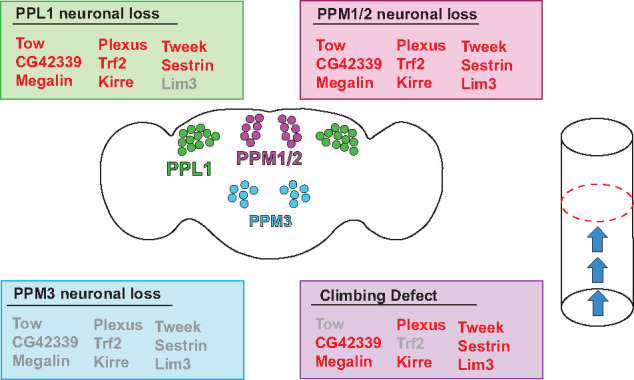
Summary of phenotypic results for each candidate gene. Candidate genes found from this screen are listed under each phenotype examined in this study. Genes that showed a defect in each phenotype (either by mutant analysis and/or RNAi knockdown) are listed in red. Genes that did not have an effect on a particular phenotype are listed in gray.

We successfully performed an unbiased genome-wide screen using 201 wild-caught DGRP lines (MacKay *et al.* 2012). We found nine top associated genes containing SNPs associated with the loss of DA neurons. We functionally validated these nine genes through RNAi knockdown, mutagenesis, and behavioral testing. We found neurodegeneration in the PPL1 and PPM1/2 clusters from both mutagenesis and RNAi knockdown. While we saw no neurodegeneration occur in the PPM3 cluster for both experimental conditions as well. This neurodegeneration was then recapitulated with a decline in locomotor function in select genes (Figure 7, Supplementary Table S5). Future studies aimed to further characterize these genes in order to understand the involvement each have in maintaining DA neurons. This characterization of these genes, may further help identify the mechanism of DA neuron longevity to eventually provide research into treatments for those with PD.

### Anatomical changes to DA neurons

Results from our screen suggest that genes associated with alterations in neuroanatomy could render DA neurons vulnerable. The top associated gene on our list, *target of wingless* (*tow*), has primarily been studied in the developing wing disc of *Drosophila*, where it acts as a repressor of the hedgehog signaling pathway (Ayers *et al.* 2009). Additionally, *tow* was shown to act downstream of both *frizzled* and *disheveled* in the planar cell polarity (PCP) signaling pathway (Chung *et al.* 2007). As the PCP signaling pathway is crucial for axon targeting in *Drosophila* mushroom body neurons (Gombos *et al.* 2015), it is possible that *tow* also regulates similar processes in DA neurons.

Another candidate gene that may regulate the anatomy of DA neurons is *TATA box binding protein-related factor 2* (*Trf2*), which encodes a core promoter recognition factor (Rabenstein *et al.* 1999). Recent evidence demonstrated that *Trf2* dominantly modifies *fruitless* activity. Knockdown *Trf2* in sexually dimorphic mAL neurons resulted in the loss of neurite formation and subsequent deficits to male courtship behavior (Chowdhury *et al.* 2017). Whether similar anatomical changes are seen in DA neurons and whether such changes render these neurons vulnerable to degeneration will be interesting to investigate in future studies.

### Alterations to synaptic function

Plexus is a nuclear matrix protein with a known role in regulating wing development (Matakatsu *et al.* 1999). However, it was more recently identified as a putative synaptic gene by transcriptomic analysis (Pazos Obregón *et al.* 2015). Since synaptic dysfunction is among the earliest known hallmarks of PD (Reeve *et al.* 2018), investigating the role of *plexus* in DA neuron degeneration could potentially help to further characterize the earliest deficits in this disease. Further evidence for the role of *plexus* in neurodegeneration comes from a screen to identify modifiers of *Drosophila* photoreceptor degeneration in a model of Spinocerebellar Ataxia 8 (SCA8) (Mutsuddi *et al.* 2004), where *px* was found to enhance neurodegeneration. Thus, despite the fact that *plexus* does not have a direct human homolog, it is potentially involved in processes that are highly associated with neurodegenerative diseases.

*Kirre* encodes a transmembrane protein belonging to the immunoglobulin superfamily that is required for myoblast fusion (Ruiz-Gómez *et al.* 2000). Interestingly, *Kirre* is also critical for the formation of synaptic connections in the *Drosophila* visual system (Sugie *et al.* 2010; Lüthy *et al.* 2014). Additionally, previous work demonstrated that *SYG-1*, a homolog to *Kirre* in *Caenorhabditis elegans*, is required for proper synaptic specificity (Shen *et al.* 2004). Thus, it will be interesting to determine whether the loss of *Kirre* function impairs the development or maintenance of synaptic connections of DA neurons in the *Drosophila* brain.

Finally, *tweek* plays a major role in the endocytosis of synaptic vesicles. Previous research demonstrates that *tweek* mutants cannot maintain vesicle release upon rapid stimulation, suggesting that *tweek* is specifically involved in synaptic vesicle recycling (Verstreken *et al.* 2009). As defects in synaptic vesicle cycling are prevalent in several models of Amyotrophic Lateral Sclerosis (ALS) (Coyne *et al.* 2017), it will be interesting to determine whether a similar defect results in the degeneration of DA neurons.

### Interactions with the immune response

There is currently very limited data regarding mutations in the uncharacterized gene *CG42339*. Bioinformatic analysis suggests that the protein encoded by this gene is involved in scavenger receptor activity, and transcripts of this gene are increased upon infection of *Drosophila* with Nora virus (Lopez *et al.* 2018). This may indicate that *CG42339* is somehow associated with phagocytes. The observation that RNAi-mediated knockdown of *CG42339* in DA neurons does not recapitulate the mutant phenotypes could be explained by several possibilities. First, it is possible that the extent of knockdown using RNAi is not sufficient to produce a noticeable phenotype. This could be addressed by the generation of additional independent RNAi transgenes and quantitative analysis of the knockdown. An alternative possibility is that the available mutations in *CG42339* are not loss of function, and would thus not be expected to recapitulate RNAi-mediated knockdown. Finally, it is possible that this gene is required in cells in addition to, or instead of, the DA neurons that are analyzed in this study. There is a strong link between activation of the immune system and neurodegeneration (Cao *et al.* 2013), and identifying the role of *CG42339* in the loss of DA neurons could provide further mechanistic details regarding this relationship.

### Cellular maintenance

One overlapping theme that was identified from our list of candidate genes is cellular maintenance. As neurons are particularly vulnerable to oxidative stress and defects in degradative processes including autophagy, several of our identified genes could be associated with DA neuron vulnerability through roles in these processes.

Sestrins, for example, are well-conserved yet poorly characterized proteins that accumulate in cells undergoing stress. In *Drosophila*, loss of *sestrin* function results in the misregulation of Target of Rapamycin signaling and subsequent age-related pathologies (Lee *et al.* 2010). Among the defects uncovered in *sestrin* mutants was impaired mitochondrial morphology. Mitochondrial dysfunction is strongly associated with an accumulation of Reactive Oxygen Species (Yen and Klionsky 2008), a stressor can severely disrupt the function of DA neurons. It is certainly possible that a similar type of defect is responsible for the loss of DA neurons upon loss of *sestrin* function in our study.

*Lim3*, which encodes a transcription factor that determines neuronal identity, also plays a crucial role in mitochondrial function. Knockdown of *Lim3* in Drosophila during early development results in mitochondrial dysfunction, ROS accumulation, and shortened lifespan in adults. In addition, several identified *Lim3* target genes are associated with mitochondrial activity (Rybina *et al.* 2019).

Finally, *megalin* encodes a multi-ligand endocytic receptor with role in cuticle pigmentation in Drosophila (Riedel *et al.* 2011). Studies in mice, however, found that *megalin* is also required for the transport of lysosomal enzymes (Nielsen *et al.* 2013). This suggests that mutations in *megalin* could impair the degradative machinery of neurons. Impairment of autophagy, for example, has a strong link to neurodegeneration (Lee *et al.* 2019). Thus, it is possible that a defect in lysosomal degradation underlies the loss of DA neurons in *Drosophila*.

Although locomotor impairment accompanied DA neuron loss in several of the conditions we examined, we also noticed cases where one phenotype was present without the other. For cases in which DA neuron loss is not coupled with a climbing deficit, one possibility is that the neurons lost in these conditions are not required for climbing ability. While some DA neurons are required for locomotor ability (Pendleton *et al.* 2002), others have defined roles in sleep and arousal (Liu *et al.* 2012), courtship behavior (Neckameyer 1998), and olfactory memory (Schwaerzel *et al.* 2003). For cases in which a climbing defect is found without a corresponding loss of DA neurons, it is possible that climbing ability could be impacted without DA neuron loss. As with most behaviors, climbing certainly requires the coordination of many different cell types. Thus, locomotor impairments could be present under these conditions due to impairments of other cell types.

Based on our results shown here, we believe that further investigation of each of these nine candidate genes will be helpful in identifying specific mechanisms that render DA neurons vulnerable.

### Funding

This research was supported by the National Institutes of Health (R01 NS110727 to DTB). The funders had no role in study design, data collection and interpretation, or the decision to submit the work for publication.

### Conflicts of interest

None declared.
